# Health in Buffalo

**Published:** 1852-09

**Authors:** 


*Health in Buffalo.* — Epidemic cholera has prevailed, to some extent, in this city during the last two months. For the most part, it has been confined to the improvident and reckless portion of the community, comparatively few cases having occurred among the better classes. The sections of the city in which the disease has most prevailed, are low, and were formerly marshy. It has been observed, also, that, in these sections, cases were most frequent in houses, or blocks, over-crowded with inmates. In one street the disease suddenly broke out in several families coincidently with an excavation through the street for the purpose of laying water pipes; and its development in this situation is attributed by Prof. Hamilton, and others, to emanations from the upturned soil, acting in conjunction with the unknown special cause which is essential to the production of the disease. The reader will find in the article by Prof. H., on this subject, in the present number, a report of the facts upon which this opinion is based. The considerations adduced by Prof. H. merit careful attention. It is clearly enough established that the special cause of cholera, whatever it may be, rarely acts with sufficient efficiency to produce the disease, unless auxiliary causes be superadded. Cholera may thus be said to depend usually on local causes. These local causes, it is to be presumed, are diverse in their character, and generally several are combined. The most important are insalubrity of situation, over crowding, intemperance in food and drink, over exertion, mental excitement, fear, etc., etc. Notwithstanding the great amount that has been written on this subject, a collection of recorded facts embracing all the appreciable circumstances attending the origin of the disease in particular districts, and in individual cases, is still a desideratum. With adequate date of this description, it might be practicable to deduce, not only the influence of combined local causes, but their relative agency separately.

The sanitary reports of the Registrar General of Great Britain during the years that the cholera prevailed in England and Wales, have lately been analyzed, and some important results determined, an account of which may be found in the Brit. and For. Med.-Chir. Rev., July, 1852. We allude to them thus briefly at this time, as it is our intention to transfer a digest of the important conclusions arrived at, in a subsequent number.

With respect to the number of cases that have occurred in this city, and the ratio of fatality, we are without definite information. The Board of Health did not begin to receive reports until some time after the disease made its appearance; and they have since adopted the silent policy — that is, no bulletins have been issued. Some cases are still occurring daily. After the cessation of the epidemic we shall endeavor to obtain the facts relating to its progress and history which the city records furnish.

Aside from cholera, the ordinary diseases of the season do not appear to be more frequent or severe than usual. Dysentery prevails to some extent, but, so far as our observations extend, the type of this disease is not malignant.

